# The effect of a periodontal intervention on cardiovascular risk markers in Indigenous Australians with periodontal disease: the PerioCardio study

**DOI:** 10.1186/1471-2458-11-729

**Published:** 2011-09-26

**Authors:** Michael R Skilton, Louise J Maple-Brown, Kostas Kapellas, David S Celermajer, Mark Bartold, Alex Brown, Kerin O'Dea, Gary D Slade, Lisa M Jamieson

**Affiliations:** 1 Boden Institute of Obesity, Nutrition, Exercise and Eating Disorders, University of Sydney, Sydney, Australia; 2 Menzies School of Health Research, Charles Darwin University, Darwin, Australia; 3 Division of Medicine, Royal Darwin Hospital, Darwin, Australia; 4 Australian Research Centre for Population Oral Health, School of Dentistry, University of Adelaide, Adelaide, Australia; 5 Department of Medicine, University of Sydney, Sydney, Australia; 6 Colgate Australian Clinical Dental Research Centre, School of Dentistry, University of Adelaide, Adelaide, Australia; 7 Baker IDI Heart and Diabetes Institute, Alice Springs, Australia; 8 Sansom Institute for Health Research, UniSA, Adelaide, Australia; 9 Department of Dental Ecology, University of North Carolina at Chapel Hill, USA

## Abstract

**Background:**

Indigenous Australians experience an overwhelming burden of chronic disease, including cardiovascular diseases. Periodontal disease (inflammation of the tissues surrounding teeth) is also widespread, and may contribute to the risk of cardiovascular diseases via pathogenic inflammatory pathways. This study will assess measures of vascular health and inflammation in Indigenous Australian adults with periodontal disease, and determine if intensive periodontal therapy improves these measures over a 12 month follow-up. The aims of the study are: (i) to determine whether there is a dose response relationship between extent and severity of periodontal disease and measures of vascular health and inflammation among Indigenous Australian adults with moderate to severe periodontal disease; and (ii) to determine the effects of periodontal treatment on changes in measures of vascular health and inflammation in a cohort of Indigenous Australians.

**Methods/Design:**

This study will be a randomised, controlled trial, with predominantly blinded assessment of outcome measures and blinded statistical analysis. All participants will receive the periodontal intervention benefits (with the intervention delayed 12 months in participants who are randomised to the control arm). Participants will be Indigenous adults aged ≥25 years from urban centres within the Top End of the Northern Territory, Australia. Participants assessed to have moderate or severe periodontal disease will be randomised to the study's intervention or control arm. The intervention involves intensive removal of subgingival and supragingival calculus and plaque biofilm by scaling and root-planing. Study visits at baseline, 3 and 12 months, will incorporate questionnaires, non-fasting blood and urine samples, body measurements, blood pressure, periodontal assessment and non-invasive measures of vascular health (pulse wave velocity and carotid intima-media thickness). Primary outcome measures are pulse wave velocity and carotid intima-media thickness.

**Discussion:**

The study will assess the periodontal-cardiovascular disease relationship among Indigenous Australian adults with periodontal disease, and the effectiveness of an intervention aimed at improving periodontal and cardiovascular health. Efforts to understand and improve Indigenous oral health and cardiovascular risk may serve as an important means of reducing the gap between Indigenous and non-Indigenous health in Australia.

**Trial Registration:**

Australia and New Zealand Clinical Trials Register (ANZCTR): ACTRN12610000817044

## Background

### The health of Indigenous Australians

Indigenous Australians are disadvantaged on almost every health and social indicator relative to their non-Indigenous counterparts. They have 15-20 years shorter life expectancy, much higher levels of cardiovascular disease, diabetes and other chronic conditions including poor oral health status, and are more likely to experience disability and reduced quality of life due to ill health [1-4]. They also have a high burden of infectious diseases alongside significant rates of metabolic risk [5].

### Cardiovascular disease

Cardiovascular diseases are a leading cause of morbidity and mortality in developed countries. The disease process that underlies the majority of cardiovascular events is atherosclerosis, an inflammatory disease of the blood vessel wall. The earliest physical evidence of atherosclerosis are fatty streaks, which are typically present in childhood. In the presence of arterial endothelial dysfunction, which is involved in the initiation and progression of atherosclerosis, these early lesions progress through to complex atheromatous lesions in adulthood, finally resulting in occlusion, plaque rupture and ischaemic events [6].

### Periodontal disease

Periodontal disease is inflammation of the tissues surrounding teeth and results from a complex interplay between bacteria and host risk factors such as long-term smoking, poor oral hygiene, poorly controlled diabetes, stress and genetic predisposition [7]. Not only have periodontal organisms adapted to survive within an environment that is constantly besieged by host defences, but they flourish in the presence of inflammation, enabling their capacity to invade host tissues and gain direct access to the circulation [8]. Repeated bacteremias and endotoxemias are characteristic of periodontal infection, and periodontal organisms have been found to co-localise within atheromatous plaques [9]. The constant exposure of the vasculature to these pathogens provides an opportunity for endothelial inflammatory activation and functional impairment. Clinically, periodontal disease manifests as deepening of the epithelial attachment around teeth, loss of periodontal attachment and, ultimately, tooth loosening.

Periodontal disease is widespread and poses a substantial problem among Australian Aboriginal populations. In Australia's second National Survey of Adult Oral Health (NSAOH), mild forms of periodontal disease were reported to affect 20 percent of the adult population, while more severe forms affected about 1 in 40 adults [10], increasing to 3 in 10 adults when the Indigenous population were considered in isolation. Similarly, Endean and colleagues reported that among Aboriginal adults seeking dental care in several Central Australian remote communities, 30.2 percent had a history of this condition [11]. The prevalence of tooth loss due to periodontal disease among Aboriginal populations is also high [12].

**Figure 1:**
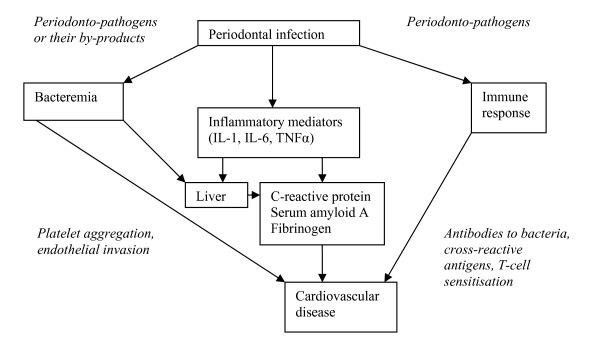
**Conceptual model of periodontal disease and cardiovascular surrogate endpoint response**.

Periodontal disease has been associated with atherosclerosis [13], cardiovascular disease [14], diabetes [15], pre-term low birth weight [16], stroke [17], and premature death [18]. Accordingly, periodontal disease may account for a portion of the risk for cardiovascular disease via a shared pathogenic underlying inflammatory response (figure 1)[8].

### Periodontal therapy decreases systemic inflammation and improves endothelial function

Treating periodontal disease results in a functional improvement in cardiovascular status [19-22]. These studies are consistent with the concept that periodontal disease may be an important source of infectious and inflammatory vascular stress, and that periodontal therapy may be of particular clinical relevance in populations with high prevalence of both periodontal disease and cardiovascular disease.

### Aims & hypotheses

The PerioCardio study will assess measures of vascular health and inflammation in Indigenous Australian adults with periodontal disease, and determine if intensive periodontal therapy improves these measures over a 12 month follow-up. The specific aims and hypotheses are:

Aim 1: To describe the extent and severity of measures of vascular health and inflammation in Indigenous Australian adults with moderate or severe periodontal disease.

Hypothesis: The extent and severity of cardiovascular surrogate endpoints in an Indigenous population with periodontal disease will be high.

Aim 2: To determine whether there is a dose-response relationship between extent and severity of periodontal disease and measures of vascular health and inflammation among Indigenous Australian adults.

Hypothesis: The extent and severity of periodontal disease has a dose-response relationship with vascular health and inflammatory measures.

Aim 3: To determine whether periodontal treatment influences vascular health and inflammation in Indigenous Australians with moderate to severe periodontal disease.

Hypothesis: Periodontal therapy will improve vascular health and inflammatory markers in Indigenous Australians.

## Methods/design

### Study design and overview

**Figure 2:**
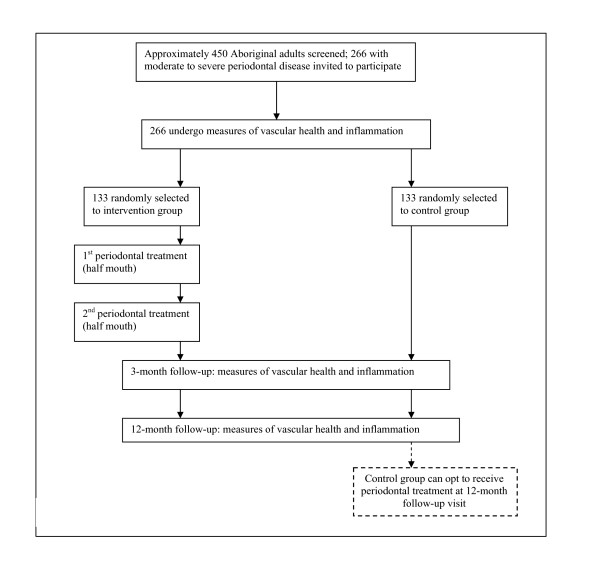
**Study plan schema**.

This will be a randomised, controlled trial, with predominantly blinded measurement of cardiovascular endpoints, and blinded statistical analysis. All participants will receive, or be offered, the periodontal intervention benefits. A schema outlining the study design is presented in Figure 2. The PerioCardio study has been registered with an international clinical trials registry (ANZCTR; ACTRN12610000817044), and will be reported as per the CONSORT statement. Recruitment for the study commenced in June 2010.

### Setting & location

Participants will be recruited from urban centres within the Top End of the Northern Territory, Australia, facilitated through the following Aboriginal Medical Services or health facilities: Danila Dilba Health Services (Darwin and Palmerston), Bagot Community Health Centre (Darwin), Royal Darwin Hospital, Darwin Dental Centre (Northern Territory Oral Health Services, Department of Health), Northern Territory Correctional Services (Darwin), and Wurli Wurlinjang (Katherine).

### Participants and recruitment

Participants will be Indigenous Australians aged ≥25 years that have lived in their current location for ≥2 years and who plan to live at their current location for the next 2 years. Those participants who have moderate or severe periodontal disease will be randomised to the intervention or control arm of the study. Those participants without periodontal disease, or only mild periodontal disease, will not be included in the trial. Baseline questionnaires will, however, be assessed in these participants in order to study the social, demographic, lifestyle and general health correlates of periodontal disease in Indigenous Australians.

Exclusion criteria include: a history of rheumatic heart disease, prior myocardial infarction, stroke or coronary revascularisation, other cardiac conditions requiring antibiotic prophylaxis for prevention of subacute bacterial endocarditis, obvious endodontic lesions, other sources of oral infection, treatment for periodontal disease within the previous six months.

Feedback to participants will be provided at the end of each examination. Participants with unexpected abnormal results will be referred to an appropriate health care provider. In addition, written results will be provided in a plain language and culturally appropriate manner to individual participants and to their nominated primary health care provider. Whenever possible, participants with abnormal results will be contacted by telephone or in person. The results packet will then be mailed or hand-delivered, as appropriate. A $50 gift voucher to a local store will be provided to participants at the end of the examination or with their results as a gift of appreciation for their participation in the study.

### Funding

Funding was provided by the National Health and Medical Research Council of Australia (NHMRC, project grant #627100).

### Ethics

The study was approved by the joint Menzies School of Health Research - Northern Territory Department of Health Human Research Ethics Committee. The project was considered and approved by both the Aboriginal sub-committee, which has absolute right of veto, and by the main committee. The study was also approved by the Central Australian Human Research Ethics Committee, Northern Territory Correctional Services Research Committee, University of Adelaide Human Research Ethics Committee, and the Aboriginal Health Council of South Australia.

### Staff

Three people are employed on contracts for the course of the study. Of these, two are Indigenous and one is non-Indigenous. Additional people are employed on a casual basis during visits to discrete locations or communities; these community members act as local facilitators. One post-graduate research student also participates in the study and is substantially involved in data collection, taking on the role of project manager. The majority of staff members have prior health qualifications and experience: one is an Aboriginal Health Worker, one has overseas dental qualifications and the post-graduate research student is an oral health therapist.

### Consent

Potential participants are provided with written information about the study, face-to-face discussion with a staff member, and given an opportunity to ask questions. If required, an interpreter explains all information relating to the study. Those who indicate that they wish to participate are then asked to complete and sign a consent form. Consent is obtained using the NHMRC Guidelines for Ethical Conduct in Aboriginal and Torres Strait Islander Health Research. All participants are informed that their participation is voluntary and that they can refuse or withdraw from participating and need give no reason or justification for their decision and that it will not affect their medical or dental care. Participants are asked to provide separate consent for various elements of the study, including: vascular assessments, blood and urine samples, body measurements, blood pressure and questionnaires. They are also asked whether they want a report sent to their primary health care provider and whether their blood and urine samples can be stored at Menzies School of Health Research for 3 years.

### Screening for periodontal disease

Following confirmation of contact details and eligibility, and after obtaining informed consent, a participant will be screened for periodontal disease, using the US Centers for Disease Control and Prevention and the American Academy of Periodontology definitions [23]. Thus, a case of moderate periodontitis is considered as the presence of either two sites between adjacent teeth with ≥4 mm attachment loss or at least two such sites with ≥5 mm pockets, and severe periodontitis as having at least two sites between adjacent teeth with ≥6 mm attachment loss and at least one pocket ≥5 mm.

### Randomisation

After screening for periodontal disease, those with moderate or severe periodontal disease are randomised on a 1:1 basis to either the treatment or control group. A computer generated permuted block randomisation sequence is used, stratified by recruitment site (Darwin/Palmerston, Katherine).

### Intervention

Individuals randomised into the 'treatment group' undergo a periodontal intervention based on the technique described previously [20]. This involves intensive removal of subgingival and supragingival calculus and plaque biofilm by scaling and root-planing. Participants are offered local anaesthesia prior to the procedure. The procedures are carried out with the use of Hu Friedy hand scalers and a piezoelectric ultrasonic scaler with universal tips. Dental extraction of teeth that cannot be saved is not performed as part of the treatment protocol. The intervention is performed by two oral health practitioners who have received additional training with these techniques. The periodontal intervention occurs during a single untimed visit to the study clinic.

Oral hygiene instruction and an oral hygiene pack containing a toothbrush and tooth paste are provided to all participants at the baseline visit. Information concerning dental extractions during the course of the study is recorded.

### Participant involvement

Each participant randomised will attend the study clinic a total of 3 times (figure 2).

Periodontal treatment will be offered to those in the control group at 12 months post-randomisation, to ensure that all participants are able to receive the oral health benefits of the periodontal treatment. The periodontal treatment offered to participants in this study is beyond that which forms routine treatment within the government funded system. Participants are not restricted from seeking periodontal treatment during the study through the private health system.

### Data collection techniques

#### Dental assessment

including tooth presence, caries experience, periodontal destruction, gingivitis and calculus, plaque, oral mucosal lesions and trauma.

#### Clinical and anthropometric measures

Blood pressure is measured while seated using an automated device (Welch Allyn Medical Products, Skaneateles Falls, USA). Three measurements of systolic and diastolic pressure are made, with three minutes between readings. The mean values of the final two recordings will be used in statistical analysis.

Height is measured to the nearest 0.1 cm using a wall-mounted stadiometer.

Weight is measured to the nearest 0.1 kilogram, using a portable weight scale (Tanita HD-351, Arlington Heights, USA) with participants lightly clothed. Two separate measurements of weight are recorded and if they differ, then a third measurement of weight is taken.

Waist and hip circumference are measured to the nearest 0.1 centimetre using a 2-metre non-stretch flexible steel tape (Model W606PM Lufkin, USA), as previously described [24].

#### Common carotid intima-media thickness (IMT) and M-mode imaging

External ultrasound of the common carotid artery is performed with the patient lying in a supine position, neck slightly extended and the head tilted away from the side being scanned whilst connected to 3-lead ECG, to monitor cardiac cycle. Bilateral longitudinal images of the carotid bulb and common carotid artery, up to 15 mm proximal to the bulb, are obtained using a portable ultrasound (Sonosite MicroMaxx, Bothell, USA), with a 10-5 MHz linear array transducer. Loops are obtained of each artery, and stored in a digital format for batch analysis by an observer blinded to participant group, characteristics and study visit. Measurement of carotid IMT will be performed on the far wall of the common carotid artery at end diastole, within 10 mm proximal to the carotid bulb, on two consecutive cardiac cycles.

M-mode imaging of the arterial diameter during the cardiac cycle is used to assess regional stiffness of the common carotid artery. The area of interest for the stiffness measure is between 10 mm and 15 mm proximal to the bulb. Loops of each side are obtained, ensuring that both the near and far walls are clearly visible and the artery is perpendicular to the angle of insonation. Internal diameter of the artery during systole and end-diastole will be measured over three cardiac cycles to generate the following indices: pressure-strain elastic modulus, Young's modulus, cross-sectional compliance, distensibility coefficient.

#### Pulse wave velocity (PWV)

PWV is measured between the common carotid and dorsalis pedis arteries using applanation tonometry (SphygmoCor-PVMx device, AtCor Medical, Sydney, Australia), with participants in a supine position for at least 10 minutes prior to commencement of measures. The common carotid artery is located via lateral palpation along the thyroid cartilage while the dorsalis pedis artery is located between the first and second metatarsals on the dorsal surface of the foot. A pressure tonometer is placed transcutaneously above the corresponding artery to record the pulse pressure waveform while simultaneously recording the ECG signal, which provides an R-wave timing reference. The arteries are assessed consecutively. The resultant PWV score is calculated via computer algorithm as the mean time difference between the R-wave and the pressure wave per heart/pulse beat and the arterial path length between the two recording sites. To correct against measurement error, the distance from the common carotid location and the sternal notch will be subtracted from the distance between the sternal notch and the dorsalis pedis site. Data quality control for this measurement requires that standard deviation not exceed 10%. In the instances that this occurs, PWV will be remeasured. Measurement of PWV is undertaken automatically by the acquisition machine, and as such the observer will not be blinded to study participant characteristics.

#### Blood and urine derived measures of cardiovascular risk

non-fasting blood and urine samples will be collected to assess established measures of cardiovascular risk, inflammation and vascular health, including glycosylated haemoglobin (HbA_1c_), lipids and lipoproteins (total cholesterol, high-density lipoprotein cholesterol, apolipoprotein A-1 and apolipoprotein B), high sensitivity C-reactive protein (hs-CRP), interleukin-6 (IL-6), asymmetric dimethylarginine (ADMA), and urine albumin to creatinine ratio.

Collected blood samples are centrifuged for 10 min at 1300 g within 30 minutes of collection. If unable to be centrifuged immediately, blood is stored in a fridge or on ice packs in a cool-box until centrifugation a maximum of 2-hours after collection. Following centrifugation, samples are transported on ice to be processed by Westerns Pathology, Darwin for lipids and HbA1c. Total cholesterol is assayed by enzymatic methods, and high-density lipoprotein cholesterol measured directly, with an ADVIA chemistry system (Siemens, Tarrytown, USA). HbA1c is determined by turbidimetric inhibition immunoassay with a COBAS INTEGRA (Roche Diagnostics, Indianapolis, USA). Other samples are stored at -80 degree Celsius. For samples collected in remote locations, storage for transportation is either on dry ice or in liquid nitrogen ("Biological Shipper", CryoPak Series, Taylor-Wharton, USA). Samples are stored for analysis at a later date for hs-CRP, IL-6, ADMA, apolipoproteins and glucose.

#### Self-administered questionnaires

Self-reported information pertaining to socio-demographic, oral health behaviours and general health factors is gathered at baseline, with the assistance of study personnel as required.

Socio-demographic details include age, sex, Aboriginal/Torres Strait Islander status, grandparental Indigenous status, education level, employment, English-as-first-language, house ownership, number of children, number of people who stayed in house the previous night and car ownership.

Oral health covariates include periodontal status and a self-report item pertaining to whether or not participants think they have gum disease.

General health covariates include behaviours such as smoking, current medication, current diagnoses (for diabetes etc), status of current diagnoses (controlled, uncontrolled etc) and stress (measured by the Kessler 10+ scale; a validated measure of distress that has been validated in a national health survey for Indigenous adults) [5].

### Primary and secondary outcomes

The primary outcome measures for purposes of this investigation will be carotid IMT and PWV. Secondary outcome measures will include CRP, IL-6, ADMA, and HbA1c.

### Sample size

Based on recent studies of Indigenous adults residing in the Northern Territory, we estimate that a sample size of 144 will provide 0.80 power to detect a 10 percent difference between groups in carotid IMT progression over 12-months, and a 10 percent reduction in PWV in the intervention group compared to the control group, at *P *< 0.05. The literature indicates that it is reasonable to expect effects of this magnitude following the periodontal intervention [25,26]. Allowing for an attrition rate of 45 percent over 12 months, 266 participants are required at baseline. In NSAOH, the proportion of Aboriginal adults with periodontal disease was estimated at 30 percent [10], however, it is likely that Aboriginal participants in NSAOH are not representative of the Aboriginal population in which we are interested. There are two main reasons: i) NSAOH participants were required to complete a telephone interview (ie to have an operating telephone and be registered in the electronic white pages); and ii) NSAOH participants were required to organise their own transport and to present for a scheduled dental examination at a local dental clinic. We posit that the NSAOH estimate is an under-estimation of periodontal disease prevalence in our target population, with anecdotal evidence suggesting that the prevalence is closer to 60 percent. We have estimated the prevalence of periodontal disease in our target population as 40 percent, meaning approximately 450 Indigenous adults will need to be screened in order to obtain 266 with moderate or severe periodontal disease.

### Data handling and statistical methods

At the time of baseline assessment, data is collected on paper forms and then entered into a Microsoft Access database. Data is stored securely at Menzies School of Health Research. In brief, the plan for the analysis is:

Aim 1: descriptive statistics including mean and standard deviation for normally distributed variables, geometric mean and 95% confidence interval for non-normally distributed data, and percentages for categorical variables.

Aim 2: associations of the extent and severity of periodontal disease with measures of vascular health and inflammation from cross-sectional analysis at baseline, using correlation analysis, and multivariable regression modelling adjusted for cardiovascular risk factors.

Aim 3: intention to treat analysis comparing cardiovascular surrogate endpoints between the two randomised groups. Primary outcomes are carotid IMT at 12-months and PWV at both 3- and 12-months. Correlations between the changes from baseline to 3- and 12-months in carotid IMT, PWV and secondary outcomes will be determined.

Appropriate transformation of the data will be performed as required. Crude correlations will be detailed, followed by multivariable modelling. Statistical significance will be inferred at 2*P *≤ 0.05.

## Discussion

The PerioCardio study will provide answers to three key questions: (1) the extent of severity of cardiovascular surrogate endpoints in an Indigenous adult population with periodontal disease; (2) whether the extent and severity of periodontal disease correlates with measures of vascular health and inflammation in Indigenous Australian adults and; (3) whether intense periodontal therapy improves these markers of vascular health and inflammation.

Furthermore the PerioCardio study will be the first investigation examining the effectiveness of periodontal therapy in changing cardiovascular surrogate endpoint levels in an Indigenous Australian population, and the first investigation to monitor changes in cardiovascular surrogate endpoints following periodontal intervention at both 3- and 12-months.

As such, the PerioCardio study will have significance for policy and planning by providing evidence of the relationship between periodontal disease, periodontal therapy, and cardiovascular disease among Indigenous Australian adults with periodontal disease, and the effectiveness of an intervention aimed at improving periodontal and cardiovascular health in an Indigenous population. Efforts to understand and improve Indigenous oral health and cardiovascular risk may serve as an important means of reducing the gap between Indigenous and non-Indigenous health in Australia.

The findings may help raise the profile of the role of periodontal disease in cardiovascular health; thus increasing the knowledge base of those working intimately with patients with cardiovascular disease. Ultimately it is hoped that the findings might encourage greater dialogue between oral health and medical professionals so that periodontal treatment might become a routine part of care in the treatment of cardiovascular disease among Indigenous as well as non-Indigenous populations.

## Competing interests

The authors declare that they have no competing interests.

## Authors' contributions

MRS participated in study design, ethics applications, data collection, data management and manuscript preparation. LMB participated in study design, ethics applications, data collection, data management and manuscript preparation. KK participated in study design, data collection, project management, data management and manuscript preparation. DSC, MB, AB, KO, GS provided important intellectual input into the study design and revision of the manuscript. LJ drove the design of the study protocol for funding and ethics applications, coordinated data collection and data management, and participated in manuscript preparation.

All authors were involved in revising the manuscript for important intellectual content and read and approved the final manuscript.

## Pre-publication history

The pre-publication history for this paper can be accessed here:

http://www.biomedcentral.com/1471-2458/11/729/prepub
